# New Insight into the Effects of Metformin on Diabetic Retinopathy, Aging and Cancer: Nonapoptotic Cell Death, Immunosuppression, and Effects beyond the AMPK Pathway

**DOI:** 10.3390/ijms22179453

**Published:** 2021-08-31

**Authors:** Sheng-Kai Hsu, Kai-Chun Cheng, Miracle Oluebube Mgbeahuruike, Yi-Hsiung Lin, Chang-Yi Wu, Hui-Min David Wang, Chia-Hung Yen, Chien-Chih Chiu, Shwu-Jiuan Sheu

**Affiliations:** 1 Department of Biotechnology, Kaohsiung Medical University, Kaohsiung 807, Taiwan; b043100050@gmail.com (S.-K.H.); cywu@mail.nsysu.edu.tw (C.-Y.W.); 2 Department of Ophthalmology, Kaohsiung Municipal Siaogang Hospital, Kaohsiung 812, Taiwan; pington64@gmail.com; 3 Department of Ophthalmology, Kaohsiung Medical University Hospital, Kaohsiung 807, Taiwan; 4 Department of Ophthalmology, School of Medicine, College of Medicine, Kaohsiung Medical University, Kaohsiung 807, Taiwan; 5 Department of Medical Laboratory Science and Biotechnology, Kaohsiung Medical University, Kaohsiung 807, Taiwan; miraclemg16@gmail.com; 6 Division of Cardiology, Department of Internal Medicine, Kaohsiung Medical University Hospital, Kaohsiung 807, Taiwan; caminolin@gmail.com; 7 Center for Lipid Biosciences, Kaohsiung Medical University Hospital, Kaohsiung 807, Taiwan; 8 Lipid Science and Aging Research Center, Kaohsiung Medical University, Kaohsiung 807, Taiwan; 9 Department of Biological Sciences, National Sun Yat-sen University, Kaohsiung 804, Taiwan; 10 Graduate Institute of Biomedical Engineering, National Chung Hsing University, Taichung 402, Taiwan; davidw@dragon.nchu.edu.tw; 11 Graduate Institute of Natural Products, Kaohsiung Medical University, Kaohsiung 807, Taiwan; chyen@kmu.edu.tw; 12 Department of Medical Research, Kaohsiung Medical University Hospital, Kaohsiung 807, Taiwan; 13 The Graduate Institute of Medicine, Kaohsiung Medical University, Kaohsiung 807, Taiwan; 14 Center for Cancer Research, Kaohsiung Medical University, Kaohsiung 807, Taiwan

**Keywords:** metformin, biguanides, aging, nonapoptotic cell death, metastasis, immunosuppression, cancer chemoprevention

## Abstract

Under metabolic stress conditions such as hypoxia and glucose deprivation, an increase in the AMP:ATP ratio activates the AMP-activated protein kinase (AMPK) pathway, resulting in the modulation of cellular metabolism. Metformin, which is widely prescribed for type 2 diabetes mellitus (T2DM) patients, regulates blood sugar by inhibiting hepatic gluconeogenesis and promoting insulin sensitivity to facilitate glucose uptake by cells. At the molecular level, the most well-known mechanism of metformin-mediated cytoprotection is AMPK pathway activation, which modulates metabolism and protects cells from degradation or pathogenic changes, such as those related to aging and diabetic retinopathy (DR). Recently, it has been revealed that metformin acts via AMPK- and non-AMPK-mediated pathways to exert effects beyond those related to diabetes treatment that might prevent aging and ameliorate DR. This review focuses on new insights into the anticancer effects of metformin and its potential modulation of several novel types of nonapoptotic cell death, including ferroptosis, pyroptosis, and necroptosis. In addition, the antimetastatic and immunosuppressive effects of metformin and its hypothesized mechanism are also discussed, highlighting promising cancer prevention strategies for the future.

## 1. Introduction

Type 2 diabetes mellitus (T2DM) is a chronic metabolic disorder that occurs when the body’s regulation of sugar (glucose) is impaired. Metformin, a biguanide, is a widely prescribed drug for the treatment of T2DM. The mean elimination half-life of orally administered metformin is approximately 4.0 to 8.7 h, and it primarily undergoes renal excretion [1]. Owing to its hydrophilic feature, metformin does not enter hepatocytes via passive transport; hence, its uptake relies on the organic cation transporter (OCT) family [2]. The fundamental mode of action of metformin is the suppression of hepatic gluconeogenesis and the promotion of glucose uptake and insulin sensitivity by peripheral tissues [3]. There are several proposed mechanisms by which metformin controls hepatic gluconeogenesis, the most intensely studied of which is the mitochondrial action of metformin in the inhibition of complex I of the respiratory chain, which suppresses ATP production and thus suppresses cellular function [4]. Suppression of ATP production leads to an increase in ADP:ATP and the AMP:ATP ratio, which results in the activation of AMP-activated protein kinase (AMPK). Alternatively, AMPK can be activated via a lysosomal mechanism that involves glucose starvation. This pathway of AMPK activation involves the formation of a complex with the proteins Axin and late endosomal/lysosomal adaptor MAPK and mTOR activator 1 (LAMTOR1) [5].

Aging is an irreversible biological phenomenon in all organisms and contributes to several diseases, including infection, osteoarthritis, cardiovascular diseases, dementia, and even cancer [6]. There is evidence that metformin could alleviate aging by modulating reactive oxygen species (ROS) and protein homeostasis primarily through AMPK signaling [7]. The detailed mechanism will be discussed in the following sections.

Apart from the AMPK pathway, emerging evidence has reported that metformin can also regulate AMPK-independent pathways, such as autophagy, oxidative stress and ER stress, to prevent retinal cells from vascular abnormalities, apoptosis, and cell senescence, which suppresses the development of diabetic retinopathy (DR). More detailed information is also covered.

According to a statistical analysis by Kim, during a mean 5.8 years of follow-up, the incidence of cancer among patients with T2DM was 21.8 and 13.2 per 1000 person-years in the no metformin and metformin groups, respectively. This finding indicated that the administration of metformin to patients with T2DM could reduce the risk of developing cancer [8]. Additionally, a recent systematic review and meta-analysis showed that metformin reduced the risk or recurrence of gastric cancer and hepatocellular carcinoma in T2DM patients [9]. Previous studies reported that metformin could suppress cancer cell survival and proliferation and induce cell cycle arrest, autophagy, and apoptosis [10]. Despite its effect on triggering apoptosis, emerging evidence has shown that several cancer cell lines develop resistance to apoptosis. For instance, the upregulation of antiapoptotic proteins (e.g., Bax inhibitor-1 (BI-1)) has been reported in non-small-cell lung cancer (NSCLC) [11]. Consequently, bypassing apoptosis and switching to nonapoptotic cell death might be helpful for cancer treatment. Unfortunately, most cancer-related deaths (approximately 90%) are attributed to metastasis [12]. Mitigating the distant metastasis ability could effectively promote patient survival. In recent years, due to a comprehensive understanding of the mechanism of immune checkpoints, immunotherapies have played a significant role in cancer treatment [13]. However, the therapeutic effects of metformin on nonapoptotic cell death, metastasis and immunosuppression are relatively less investigated. In this review, we will elucidate the effects of metformin against aging, DR and cancer through diverse mechanisms.

## 2. Effect of Metformin on Aging

Aging is a biological phenomenon in all organisms and can be characterized by an accumulation of various deleterious changes with time associated with an increased risk of susceptibility to disease and death. These changes can be inherited or attributed to genetic defects, disease, and the environment. Although aging itself cannot be considered a disease, it is an indisputable fact that age-associated diseases are among the leading causes of death globally, primarily in industrialized countries [14]. In recent years, there has been intensive research on the antiaging effects of the hypoglycemic drug metformin, drawing much attention to its potential geroprotective effects.

Aging is a malleable process that can be accelerated or slowed in response to genetic and environmental stimulations and epigenetic modifications expressed through changes in various cellular signaling pathways and metabolic processes, including oxidative stress, telomere attrition, epigenetic alterations, proteostasis, autophagy and mitochondrial functions. This is because aging is regulated by various cellular signaling mechanisms, including the nutrient-sensing pathway, protein homeostasis, and ROS-mediated oxidative stress, which will be discussed in this section [7].

*Caenorhabditis elegans* (C. elegans) has emerged as one of the premier model systems for aging research, with metformin extending the lifespan of roundworm elegans by up to 50%, a discovery that enabled genetic dissection of the pathways necessary for metformin longevity effects [15]. The cellular energy sensor AMPK is genetically required for metformin-mediated lifespan extension in C. elegans. Additionally, AMPK upstream activating kinase liver kinase B1 (Lkb1, par-4 in the worm) and the stress-induced transcription factor skn-1/nuclear factor erythroid 2-related factor 2 (Nrf2) are required for the mediation of this effect of metformin, suggesting that the glycemic and antiaging effects of the drug have distinct mechanisms of action [16].

Metformin exerts its life-extending effects via the nutrient-sensing pathway, a major pathway that regulates nutrient uptake and signaling and thus influences many metabolic processes and promotes growth. The insulin/IGF-1 signaling (IIS) pathway, consisting of the insulin/insulin growth factor-1 (INS/IGF-1) receptor and its adaptor protein insulin receptor substrate 2 (IRS2), is key in this process [7]. Metformin inhibits the actions of IGF and IRS2 and extends lifespan; for example, mice with downregulation of IGF receptors or IRS2 experienced prolonged lifespans [14,17].

In addition to promoting longevity by modulating metabolic pathways, metformin can also extend lifespan by modulating protein homeostasis. In a previous study, reduced global progerin cells were observed in human mammary carcinoma after treatment with metformin; this effect of metformin was modulated via activation of the AMPK pathway [18]. The progerin protein is a mutated form of the nuclear envelope protein lamin A (LMNA) that induces early cellular senescence associated with increased DNA damage signaling [7]. Furthermore, mutant *LMNA* might lead to aberrant protein expression; in addition, progerin is highly involved in the pathogenesis of the rare genetic premature aging syndrome Hutchinson-Gilford progeria syndrome (HGPS) [19,20]. Metformin, mediated by the AMPK pathway, suppressed protein translation by inhibiting mammalian target of rapamycin (mTOR), which consequently results in a decrease in the phosphorylation of ribosomal protein S6 (rsP6), S6 kinase (S6K) and eIF4E-binding protein 1 (eIF4E-BP1) [14,18].

**Figure 1:**
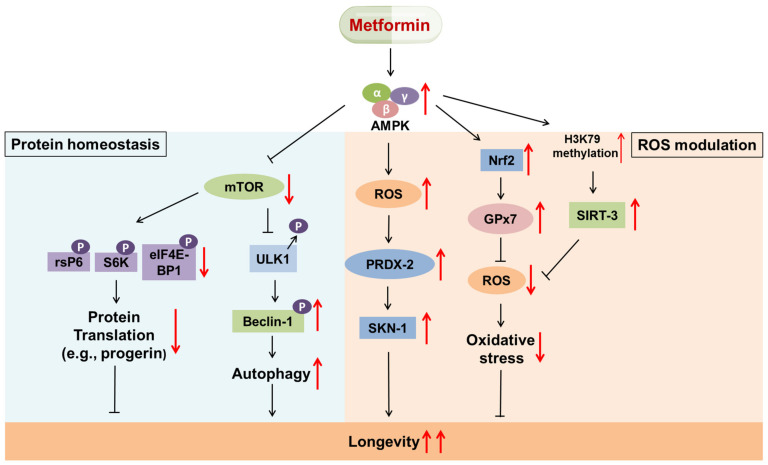
The antiaging effect of metformin. (Left panel) Metformin exerts an antiaging effect by modulating protein homeostasis. Metformin can suppress protein translation (e.g., progerin) and enhance autophagy to extend longevity via AMPK-induced inhibition of mTOR activity. It impedes the translation of aging-related proteins such as progerin through a decrease in the phosphorylation of ribosomal protein S6 (rsP6), S6 kinase (S6K) and eIF4E-binding protein 1 (eIF4E-BP1). In addition, metformin initiates the dephosphorylation of ULK1 and further results in the phosphorylation of Beclin-1, which triggers autophagy. (Right panel) Metformin exerts an antiaging effect by modulating ROS. ROS serve as a double-edged sword in the modulation of the aging process. On the one hand, AMPK-induced accumulation of ROS facilitates longevity via the PRDX-2/SKN-1 axis. On the other hand, AMPK alleviates ROS accumulation and increases longevity through the Nrf2/GPx7 axis of SIRT-3.

Alternatively, the elimination of aging-related proteins via autophagy can support protein homeostasis and thus increase cell survival. Cells utilize autophagy, an intracellular self-digestion mechanism, to dispose of cellular waste, thus promoting cell fitness, genome integrity, tissue homeostasis, and cell survival and growth under stress [21]. Metformin can induce the inhibition of mTOR activity and IIS pathway-mediated PI3K/Akt activation, which in turn leads to the dephosphorylation of Unc-51-like autophagy activating kinase 1 (ULK1) (a homolog of yeast ATG1), consequently activating ULK1 kinase to induce autophagy via the formation of a phagophore. In turn, through the phosphorylation of Beclin-1 (a homolog of yeast ATG6), ULK1 promotes the recruitment of autophagy proteins for phagophore elongation. This generates an LC3-phosphatidylethanolamine complex (LC3-PE complex) under the action of Atg5, forming autophagosomes. Autophagosomes and lysosomes eventually fuse to form autophagolysosomes, where the engulfed contents are degraded by lysosomal hydrolases [14,21]. In a study, it was observed that Atg5 overexpression markedly prolonged lifespan [22]. Furthermore, in the fruit fly *Drosophila*, metformin had an inhibitory effect on the early senescence of intestinal stem cells (ISCs). However, upon the knockdown of Atg6, an autophagy-related protein, the anti-senescence effect of metformin diminished (Figure 1) [23]. Apart from nutrient-related signaling and mTOR signaling, a recent study suggested that NAD-dependent deacetylase sirtuin-1 (SIRT-1) was positively correlated with the induction of autophagic flux. SIRT-1 stimulates forkhead box O3 (FOXO) activity via deacetylation to induce the expression of essential autophagy genes. Overall, IIS signaling suppression, mTOR inhibition and SIRT-1 upregulation contribute to autophagy induction and extended longevity [24].

ROS are inevitable toxic byproducts of cellular metabolism that can accumulate and damage cellular components such as DNA, lipids, proteins, and mitochondrial DNA (mtDNA), and the depletion of these components is associated with aging and aging-related pathologies. Accumulating evidence has suggested that metformin can delay aging by regulating ROS levels, thus preventing aging-related diseases that can result from mitochondrial dysfunction and oxidative stress. Thus, ROS serve as a double-edged sword in aging.

Recent research showed a significant increase in ROS levels in metformin-treated osteosarcoma cells [25]. Similarly, another study reported an increase in ROS in *C. elegans* after treatment with metformin and further noted that metformin treatment activated the SKN-1 transcription factor (the ortholog of mammalian Nrf2), a longevity-promoting factor, via peroxiredoxin-2 (PRDX-2). Deletion of prdx-2 resulted in a drastic decrease in lifespan in *C. elegans* after metformin treatment [26]. In addition, it has been reported that metformin can reduce ROS levels by upregulating certain antioxidants, such as endoplasmic reticulum (ER)-localized glutathione peroxidase 7 (GPx7). This previous study showed that GPx7 expression decreased senescence, while its knockout was associated with increased oxidative stress, premature senescence, and shorter lifespan. Metformin was linked to the accumulation of Nrf2, which promoted GPx7 expression. This metformin-nrf2-GPx7 pathway was also observed in worms (Figure 1) [27].

Furthermore, metformin can regulate ROS levels by upregulating SIRT-3 (a mitochondrial deacetylase) levels in the mitochondria and alleviating oxidative stress. One study found that SIRT-3 knockdown was responsible for increased oxidative stress and early senescence. Metformin-dependent AMPK activation was linked to the amplified methylation of H3K79, which in turn resulted in increased levels of SIRT-3 (Figure 1) [28]. A study reviewed by Robert suggested that the downregulation of mitochondrial SIRT-3 was positively correlated with age-associated disorders, including insulin resistance, cardiovascular diseases and neurodegeneration [29]. Another study conducted by Benigni indicated that *Sirt3*^-/-^ mice exhibited cardiac hypertrophy and fibrosis and experienced a shorter life span than wild-type mice. Conversely, the transfer of deacetylated optic atrophy 1 (OPA1), a downstream target of SIRT-3, reversed age-related cardiac disorders [30].

Metformin is also suggested to exert inhibitory effects on aging-related morbidities, such as obesity, cardiovascular diseases and neurodegenerative disorders. Regarding obesity, metformin can reverse insulin treatment-induced weight gain and reduce appetite through microbiome alterations and gut-brain axis [31]. The previous large observational studies indicated that significant decrease in the incidence of coronary artery disease (CAD) was observed in early- or late-stage DM patients treated with metformin compared to sulfonylurea monotherapy [32]. In addition, chronic hyperglycemia can result in Aβ aggregation and neuroinflammation; in contrast, metformin can reverse neuron degeneration [33]. Metformin is seen as a promising preventive agent for aging-related complications; nevertheless, the detailed mechanisms are needed further investigation.

## 3. Metformin in Diabetes and DR

DR is one of the major causes of adult vision loss in developed countries. However, the exact pathogenesis mechanism is unclear. DR, characterized by leaky vascular structures and aberrant angiogenesis, is divided into two stages: nonproliferative diabetic retinopathy (NPDR) and proliferative diabetic retinopathy (PDR). The former is primarily caused by inflammation-induced elevated vascular permeability; the latter, an advanced stage, is mainly attributed to increased neovascularization [34]. The retinal pigment epithelium (RPE) is a single layer of epithelial cells lining the posterior segment of the eye that serves a variety of important functions protecting and maintaining visual function. In patients with long-term DR, the degeneration of the RPE progresses even if the other conditions are well controlled and the retinopathy is stable. In addition to diabetes treatment, metformin has been shown to exert a protective effect on RPE cells in vitro and in vivo [35]. Recent studies have highlighted the involvement of vascular abnormalities, apoptosis, and cell senescence in the pathogenesis of DR. Furthermore, impaired autophagy has been shown to be involved in the exacerbation of DR, suggesting the important role of autophagy in preventing RPE cell apoptosis or senescence. At the molecular level, DR mechanisms can be divided into those related to AMPK-dependent and AMPK-independent pathways.

### 3.1. AMPK-Dependent Pathway

AMPK, which consists of three different subunits, serves as a key regulator of energy homeostasis in cells; furthermore, it has been shown that metformin can activate AMPK [36]. Shrikanth et al. reported that downregulation of AMPK results in the suppression of the NAD-dependent deacetylase SIRT-1, which inhibits NF-κB activation via deacetylation of its p65 subunit. Moreover, NF-κB can trigger the generation of inflammatory cytokines and upregulate vascular cell adhesion protein 1 (VCAM-1), a protein that strengthens the inflammatory response through the recruitment of inflammatory cells, subsequently leading to the increased permeability of retinal vessels [37,38]. Other evidence has indicated that the level of C1q/TNF-related protein-3 (CTRP3) (also known as CORS26, cartducin, or cartonectin) is decreased widely in patients with T2DM, although its association with DR has not been reported. CTRP3 can lead to AMPK phosphorylation and VCAM-1 downregulation. Conversely, the silencing of AMPK by siRNA does not suppress the generation of VCAM-1 induced by high hyperglycemia lipid (HGHL) levels when CTRP3 is present [39]. This indicates that intact AMPK prominently inhibits inflammation and the development of DR.

The unfolded protein response (UPR) is activated once aberrant proteins are present, which leads to the repair and clearance of abnormal proteins. The accumulation of an excess of misfolded proteins might eventually cause apoptotic cell death [40]. Previous evidence has shown a close correlation among the UPR, ROS, and autophagy [41]. The UPR modulates oxidative stress primarily via the PERK and ATF6 signaling pathways. Nrf2 serves as a key factor in antioxidation by upregulating antioxidant-related genes and interacts with Keap1, a molecule that facilitates Nrf2 degradation. PERK activation can result in the liberation of Nrf2 by changing the conformation of Kelch-like ECH-associated protein 1 (Keap1) and upregulating ATF4 to induce the generation of GSH. Both effects are conducive to removing the accumulation of oxidative substances [42,43]. It is evident that oxidative stress can not only upregulate VEGF-A (a mediator of neovascularization in DR) via the Raf/MEK/ERK1/2 axis but can also induce apoptosis via the upregulation of Bax and Bcl-2 [44,45]. ATF4 and ATF6 can activate AMPK, which causes mTOR1 inhibition and in turn promotes ULK1 activation, both of which trigger autophagy. In addition, PERK is suggested to transform LC3-I into LC3-II. X-Box binding protein 1 (xBP1), an active transcription factor induced by IRE-1, is reported to upregulate *BECN1* and *ATG,* which are important autophagy initiators [43]. Other evidence has shown that under ER stress, excessive cytosolic calcium contributes to autophagy mainly via three signaling pathways: induction of CamKK/AMPK to suppress mTOR and stimulation of AMPK to Beclin1 phosphorylation [46,47].

Müller cells (MCs) play a crucial role in the retina’s physiological function. However, apoptosis exerts a harmful effect on MCs and promotes the pathogenesis of DR. In contrast, the administration of metformin and resveratrol (a phytoalexin derived from plants) can induce AMPK activation and reverse the suppression of SIRT-1, preventing the senescence of retinal cells [48].

**Figure 2:**
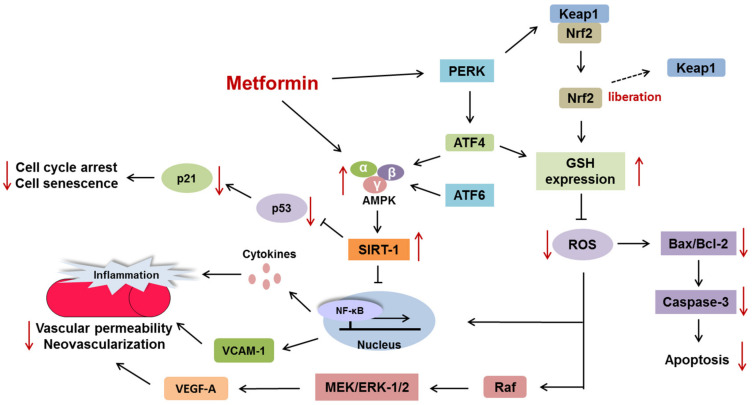
Therapeutic effects against vascular abnormalities and the apoptosis of retinal cells.

Metformin is a commonly used oral hypoglycemic agent that can exert a hypoglycemic effect by decreasing hepatic gluconeogenesis due to the activation of AMPK and the facilitation of AMPK-mediated translocation of SLC2A4/glucose transporter type 4 [5,6]. The administration of metformin is reported to inhibit inflammation, ROS generation, endothelial cell senescence, and neovascularization through the modulation of AMPK and SIRT-1, which is a potential strategy to prevent or attenuate the pathogenesis of DR (Figure 2).

### 3.2. AMPK-Independent Pathways

#### 3.2.1. Vascular Abnormalities

Autophagy is a cell survival-related process by which intracellular substances are recycled and degraded, and it has been shown to have roles in neuroprotection and DR prevention [49]. Autophagy has been proposed to serve as a double-edged sword in DR: Under mild stress, autophagy can help cells overcome adverse conditions; however, excessive autophagy leads to massive cell death and DR exacerbation [47]. Furthermore, the impairment of autophagy is also associated with the pathogenesis of DR. Lopes’ experiment showed that with exposure to hyperglycemia, Beclin-1, LC3II and p62/SQTSM1 levels are elevated in retinal MCs, which indicates autophagosome formation; however, autophagosome degradation is decreased under these conditions. In vitro and in vivo results showed that hyperglycemia promoted autophagy but increased the accumulation of p62/SQTSM1 cargo due to lysosomal dysfunction, leading to massive vascular endothelial growth factor (VEGF) release and MC death in an animal model. In addition, metformin is suggested to not only suppress the phosphorylation of VEGFR2 but also induce VEGF-A mRNA splicing to VEGF120, resulting in decreased activity between VEGFR2 and VEGF-A. This reverses the progression of DR by suppressing angiogenesis [50].

#### 3.2.2. Apoptotic Cell Death

In terms of oxidative stress, prolonged exposure to high glucose levels leads to increased ROS production due to an imbalance in metabolism [51]. For instance, Kowluru et al. indicated that diabetes can trigger oxidative stresses by epigenetic modification. Manganese superoxide dismutase (MnSOD), an important enzyme for scavengers of oxidative species, could be downregulated via H4K20me3 at the promoter of *sod2* (a gene encoding MnSOD) [52]. Chronic glucose exposure causes the upregulation of NADPH oxidase (NOX) and oxidative stress, triggering matrix metalloproteinase 9 (MMP-9) activation. MMP-9 activation leads to mitochondrial dysfunction (e.g., elevated membrane permeability and release of cytochrome c); additionally, these effects predispose retinal cells to apoptosis [53]. Cheng et al. demonstrated that metformin suppressed intracellular ROS through the upregulation of MnSOD and the downregulation of NOX2 and NOX4 in the pancreatic cancer cell lines MiaPaca and Panc-1 [54]. Hence, it is hypothesized that metformin could prevent retinal cells from ROS-induced apoptosis via the modulation of MnSOD and NOX.

Lysosomal impairment and autophagic dysfunction are early events involved in the pathogenesis of DR. Autophagy inhibitors, including 3 methyladenine (3-MA) and bafilomycin (BAF), block autophagosome formation, but the latter interferes with the fusion of lysosomes to autophagosomes. The administration of 3-MA results in the downregulation of Beclin-1 and the upregulation of p62/SQTSM1; treatment with BAF also induces the accumulation of p62/SQTSM1. In addition, both inhibitors increased the activity of caspase-8 compared to that in untreated cells. In conclusion, inhibition of the initial stage of autophagy or autophagic flux may lead to apoptosis under hyperglycemic conditions [55].

ER stress is a highly regulated mechanism involving three signaling pathways (IRE-1, PERK, and ATF6) that participates in protein quality control within cells. However, aberrant or prolonged ER stress may result in the accumulation of misfolded proteins and trigger proapoptotic cell death [40]. Similarly, ER stress plays dual roles in the pathogenesis of DR. It has been reported that the induction of PERK and CHOP, accompanied by apoptosis, is highly associated with DR pathogenesis. Furthermore, hyperglycemia-induced ER stress can also initiate the apoptosis of retinal cells via the IRE-1/JNK axis and the activation of caspase-12 and caspase-3 [56]. In contrast, the upregulation of Bip, a chaperone responsible for repairing unfolded proteins, suppresses CHOP-triggered apoptosis in retinal cells [57].

Thapsigargin (THAP), a guaianolide-type sesquiterpene lactone, can lead to ER stress-induced apoptosis by increasing ROS generation, suppressing oxidative phosphorylation, and promoting mitochondrial dysfunction. Chen et al. demonstrated that metformin could reverse these effects by suppressing CHOP-triggered apoptosis and restoring mitochondrial function, which prevents cells from undergoing apoptosis [58]. Another study demonstrated that retinal MCs treated with PERK inhibitors show decreased Beclin-1 and elevated p62/SQTSM1 expression; this support a close correlation between ER stress and autophagy [55].

#### 3.2.3. Cell Senescence

Stress-associated premature senescence (SASP) can trigger microvascular impairment and DR due to the hyperglycemia-induced upregulation of miR-34a, which has been confirmed to inhibit SIRT-1 and SOD2 (an important antioxidant in mitochondria); this leads to elevated ROS and mitochondrial dysfunction, further exacerbating the pathogenesis of DR [59]. There are two isoforms of the key urea cycle enzyme arginase, arginase 1 and arginase 2; arginase is suggested to induce intracellular ROS generation by competing with nitroxide synthase (NOS) for the substrate L-arginine [60]. Exposure to high glucose (hyperglycemia) causes the upregulation of arginase and promotes retinal endothelial cell senescence and cellular stress. In addition, an experiment conducted by Zhang indicated that hyperglycemia-triggered ROS could initiate p53 acetylation and p21 activation primarily via p300/CBP, finally resulting in endothelial senescence. Moreover, there was a significant increase in the expression of senescence-related genes, such as p21, p16INK4, and p53, in diabetic mice compared with nondiabetic mice. However, this situation can be reversed by administering the arginase inhibitor 2(S)-amino-6-boronohexanoic acid (ABH). Altogether, these findings sin indicate that arginases upregulation promote ROS accumulation under hyperglycemic conditions, contributing to endothelial senescence and DR progression [61,62].

## 4. Metformin and Cancer

### 4.1. Induction of Nonapoptotic Cell Death by Metformin

In recent decades, the biological characteristics of apoptosis have been well established. Most chemotherapies targeting the mechanism of apoptosis have achieved remarkable successes; nevertheless, emerging evidence has indicated that several cancer cell lines are chemoresistant due to defects in apoptotic cell death. For instance, the breast cancer cell lines MCF-7 and MDA-MB-231 are resistant to docetaxel owing to the upregulation of miRNA-34a and miR-141 [63,64]. Hence, bypassing apoptotic cell death mechanisms and emphasizing nonapoptotic cell death mechanisms, such as necroptosis, pyroptosis, and ferroptosis, seems to be a potential strategy to mitigate chemoresistance.

### 4.2. Necroptosis

Necroptosis is an alternative cell death primarily initiated by interactions between death receptors (TNFR superfamily) and corresponding ligands (e.g., FasL and TNF-α). Moreover, receptor-interacting serine/threonine-protein kinase 1 (RIPK1) and receptor-interacting serine/threonine-protein kinase 3 (RIPK3) are important components, and mixed lineage kinase domain-like pseudokinase (MLKL) plays a vital role in executing necroptotic cell death via pore formation on the cell membrane [65]. Furthermore, unlike apoptosis, a noninflammatory cell death mechanism, necroptosis can induce immunity through the release of damage-associated molecular patterns (DAMPs), including ATP and high mobility group box 1 (HMGB1), and chemokines [66].

**Figure 3:**
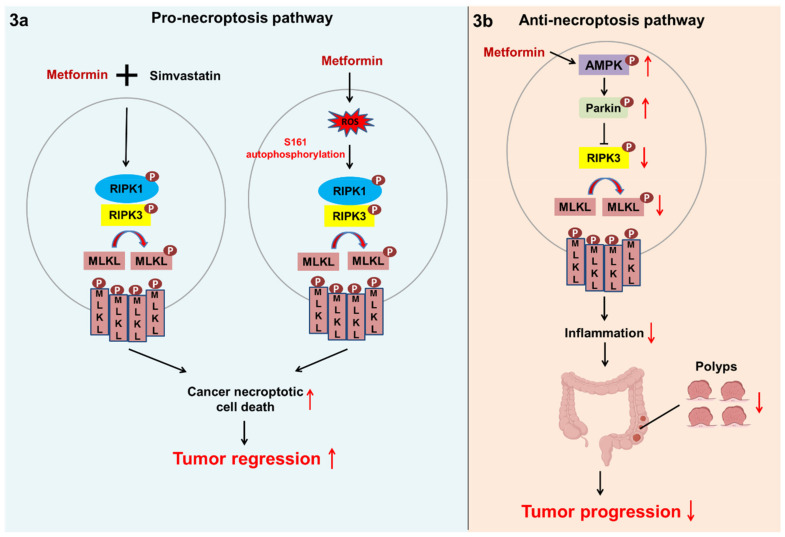
Necroptosis may be a double-edged sword in cancer progression. (**a**) In the CRPC cell line C4-2B, the combination of metformin and simvastatin upregulates RIPK1 and RIPK3, which further triggers necroptotic cell death. In the breast cancer cell line MCF-7, metformin promotes ROS generation and subsequently induces the autophosphorylation of RIPK1 at S161, leading to MLKL phosphorylation and necroptosis. (**b**) Metformin induces the AMPK-mediated phosphorylation of Parkin; activated Parkin blocks necroptosis and inflammation via the inhibition of RIPK3. This reduces the formation of polyps and suppresses the tumorigenesis of CRC.

Metformin-mediated necroptosis serves as a double-edged sword in cancer progression: metformin mediates antitumor effects by inducing necroptosis and protumor effects by promoting necroptosis-related inflammation. On the one hand, metformin can induce necroptosis in the breast cancer cell line MCF-7. On the other hand, it can promote ROS generation, leading to autophosphorylation at S161 of RIPK1 to trigger necroptosis. However, the cytotoxic effects of metformin could be alleviated through the administration of necrostatin-1 (Nec-1), a RIPK1 inhibitor [67]. Simvastatin is a statin drug and functions as an HMG-CoA reductase inhibitor widely prescribed for patients with hypercholesterolemia [68]. Babcook et al. indicated that a combination of simvastatin and metformin synergistically induces necroptosis in the metastatic castration-resistant prostate cancer (CRPC) cell line C4-2B via upregulation of RIPK1 and RIPK3 (Figure 3a). This may make CRPC more sensitive to chemotherapy [69,70]. On the other hand, Parkin (also known as PARK2) is an important E3 ubiquitin ligase that triggers RIPK3 polyubiquitination, and its mutation is associated with familial Parkinson’s disease [71]. Furthermore, Parkin deficiency brings about pancreatic tumorigenesis due to increasing spindle misorientation during mitosis [72]. A preliminary study suggested that metformin-induced phosphorylation of AMPK can activate Parkin through phosphorylation at S9. Lee et al. demonstrated that activated Parkin suppressed RIP3-mediated necroptosis and that hyperinflammation increased the number of polyps and promoted the tumorigenesis of colorectal cancer (CRC) in mouse models of azoxymethane- and dextran sulfate sodium (AOM−DSS)-induced colitis-associated colon cancer (Figure 3b) [73]. In this respect, metformin suppresses cancer progression by alleviating necroptosis-mediated inflammation.

### 4.3. Pyroptosis

Pyroptosis, a form of nonapoptotic cell death, is highly associated with immunostimulation through the gasdermin (GSDM) protein superfamily. Its morphology is characterized by membrane blebbing with bubble-like protrusions (known as pyroptotic bodies) and culminates in membrane rupture [74]. Cell swelling and pore formation are executed via the N-terminus of the gasdermin protein, which translocates to the plasma membrane, forming oligomers and pores [75]. With regard to the molecular mechanism, pyroptosis mechanisms can be classified into two pathways: the canonical pathway mediated by caspase-1 and gasdermin D (GSDMD) and the noncanonical pathway initiated by caspase-4/-5/-11 (caspase-4/-5 in humans and caspase-11 in mice) or caspase-3 [76].

**Figure 4:**
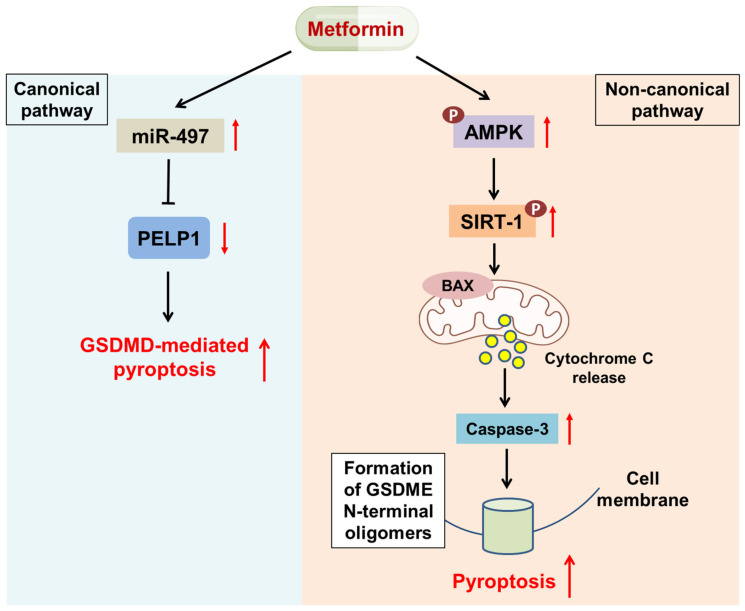
Metformin initiates canonical and noncanonical pyroptosis. Left panel: Canonical pyroptosis: metformin induces GSDMD-mediated pyroptotic cell death via the upregulation of miR-497 and the subsequent inhibition of FELP1. Right panel: Noncanonical pyroptosis: metformin triggers the activation of AMPK, which phosphorylates SIRT-1. This upregulates BAX and promotes the release of cytochrome C from mitochondria, which initiates caspase-3- and GSDME-mediated pyroptotic cell death.

Proline-, glutamic acid-, and leucine-rich protein 1 (PELP1) acts as an oncogenic protein and modulates the functions of hormone receptors [77]. PELP1 is reported to promote breast cancer cell line MCF-7 resistance to tamoxifen [78]. Moreover, PELP1 indicates a poor prognosis in patients with esophageal squamous cell carcinoma (ESCC). Wang et al. indicated that metformin could suppress PELP1 and further induce GSDMD-mediated pyroptosis via upregulation of miR-497 in the ESCC cell lines KYSE510 and KYSE140 (Figure 4) [79]. An experiment conducted by Zheng suggested that metformin can induce pyroptotic cell death via the AMPK/SIRT-1 signaling pathway. On the one hand, metformin-initiated AMPK/SIRT-1 and further phosphorylated the NF-kB p65 subunit. This resulted in BAX expression and cytochrome C release, subsequently mediating caspase-3/GSDME noncanonical pyroptosis (Figure 4). On the other hand, as a mitochondrial complex I inhibitor, metformin triggered mitochondrial dysfunction and further promoted AMPK/SIRT-1 to form positive feedback. Cell swelling and pore formation were observed in the breast cancer cell line MCF-7 and the hepatocellular carcinoma cell line HepG2 and were accompanied by increased levels of lactate dehydrogenase (LDH, an indicator of pyroptosis) [80]. In conclusion, metformin-mediated pyroptosis can bypass the resistance to apoptosis.

### 4.4. Ferroptosis

**Figure 5:**
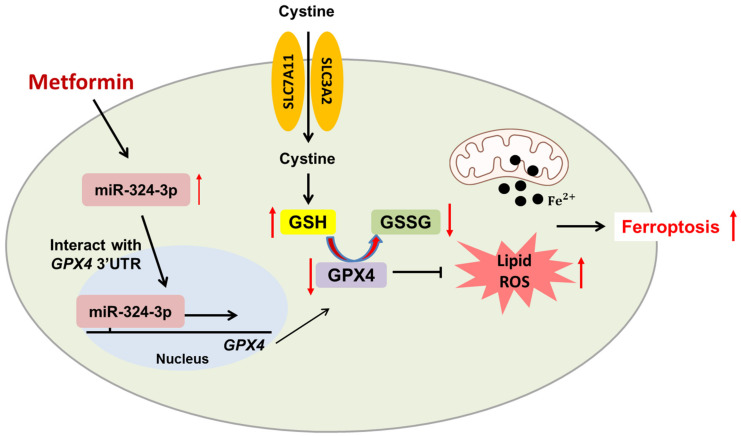
Metformin triggers ferroptosis. Metformin downregulates GPX4 by upregulating miR-324-3p; this contributes to the increased accumulation of lipid ROS and further promotes ferroptosis.

Like pyroptosis, ferroptosis is also a novel nonapoptotic cell death characterized by dependence on iron, accumulation of ROS, and lipid peroxidation. Preliminary evidence has shown that ferroptosis can be induced by immunotherapy to inhibit tumor growth. It is evident that CD8+ T cells can be restored and reactivated after anti-PD-L1 mAb treatment, which further secretes interferon-gamma (IFN-γ) to downregulate two subunits of the anti-transporter SLC3A2 and SLC7A11. Subsequently, this augments lipid peroxidation in tumor cells and triggers ferroptosis [81]. In addition, Hou and his colleagues revealed that metformin could induce ferroptosis in the triple-negative breast cancer (TNBC) cell line MDA-MB-231 via upregulation of miR-324-3p, which directly interacted with the 3’ untranslated region (UTR) of glutathione peroxidase 4 (*GPX4)* and further inhibited GPX4 (Figure 5) [82].

The LKB1/AMPK signaling pathway plays a significant role in energetic homeostasis. In addition, LKB1 is a tumor suppressor and is inactivated in several types of cancer, such as lung cancer, cervical cancer, and pancreatic cancer [83,84,85,86].

Acetyl-coenzyme A carboxylase 1 (ACC1) and fatty acid synthase (FAS) are important enzymes for fatty acid synthesis; the former converts acetyl-CoA into malonyl-CoA, and the latter transforms malonyl-CoA into palmitate. A previous study showed that LKB1 and its downstream AMPK can block ferroptosis by inhibiting the phosphorylation of ACC1 and FAS [87]. Collectively, the induction of ferroptosis in LKB1-mutated and treatment-refectory cancer cells is a promising treatment strategy. Although there is no direct evidence that ferroptosis could be suppressed by metformin-mediated phosphorylation of AMPK, the correlation between metformin and ferroptosis needs further investigation.

### 4.5. Metformin and Metastasis

Most cancer-related deaths (approximately 90%) are attributed to metastasis. Metastasis refers to the process by which cancer cells translocate from their original (primary) organ to a distant (secondary) organ. It is characterized by epithelial-mesenchymal transition (EMT) and mesenchymal-epithelial transition (MET), and both processes are necessary for cancer cells to establish new colonies. Hence, the suppression of metastasis can considerably improve the survival rate of patients [12,88].

LKB1, a serine/threonine kinase, facilitates the phosphorylation of AMPK at Thr172, which contributes to the upregulation of related genes, including those related to glycolysis and fatty acid synthesis [89]. Nevertheless, the LKB1/AMPK axis serves as a double-edged sword for cancer progression. LKB1/AMPK induces Keap1 degradation and the subsequent activation of Nrf2 under glucose starvation conditions, which in turn upregulates MMP-9 to promote the invasiveness of HepG2 hepatocellular carcinoma cells [90]. In contrast, Song and his colleagues reported that metformin can block the migration and invasion of PANC-1 pancreatic ductal adenocarcinoma cells by inducing the expression of LKB1. Then, LKB1 can promote the interaction between Snail (a vital protein for EMT) and the E3 ligase FBXL14, which results in the proteasomal degradation of Snail [91]. In addition, a preliminary study showed that metformin can block the invasion and metastasis of the SMAD4-mutant pancreatic ductal adenocarcinoma (PDAC) cell lines T3M4 and BxPC3. SMAD4 serves as a significant suppressor of hepatocyte nuclear factor 4 gamma (HNF4G), which modulates cell-cell junctions. When SMAD4 is mutated, metformin can activate AMPK and in turn phosphorylate HNF4G, which contributes to the degradation of HNF4G and reduces the metastasis of PDAC [92].

Glioma-associated oncogene homolog 1 (GLI1) was originally identified in glioma and reported to be involved in EMT and chemoresistance [93]. The experiment conducted by Corte indicated that the combination of metformin and MEK inhibitors (e.g., selumetinib) suppressed metastasis in the NSCLC cell lines H1299 and H1975 by downregulating GLI1, independent of Kras mutation status. In addition, this combination therapy resulted in reduced MMP-2 and MMP-9 levels due to interference with NF-κB binding to their promoters [94].

Dipeptidyl peptidase-4 (DPP-4) inhibitors are antidiabetic drugs that promote incretin levels, which can further increase insulin levels and decrease glycogen levels [95]. Although DPP-4 exerts negative effects on glucose homeostasis, it plays a vital role in suppressing invasion and metastasis by attenuating the CXCL12/CXCR4 axis. Kawakita and his colleagues indicated that metformin can inhibit DPP-4 inhibitor (KR62436)-induced metastasis of the TNBC cell line MDA-MB231 through the suppression of mTOR, which is accompanied by the downregulation of α-SMA and the upregulation of E-cadherin [96].

There is a correlation between EMT and resistance to chemotherapy and even immunotherapy [97,98]. EMT is attributed to several factors, such as PARP inhibitors (PARPis) resistance. PARPis are prescribed for breast cancer patients with mutated *BRAC* to trigger synthetic lethality, but the overall response rate (ORR) does not align with the predictions. Han et al. suggested that the phosphorylation of Akt at S473 leads to the upregulation of EMT-related markers, such as N-cadherin and vimentin, and programmed death-ligand 1 (PD-L1), in the PARPi-resistant TNBC cell line HCC1806. Metformin can sensitize HCC1806 to CD8+ T cell cytotoxicity and block EMT by reversing the phosphorylation of Akt [99]. Another study conducted by Park reported that the combination of metformin and phenformin, another antidiabetic drug, can not only promote apoptosis by suppressing the effects of phosphorylated STAT3 on antiapoptotic proteins, including XIAP, cIAP1 and survivin, but also attenuate the expression of EMT proteins, such as Twist, Snail, ZEB1 and vimentin, through the TGFBR2/Smad2 signaling pathway in the rectal cancer cell lines SW837 and SW1463 (resistant to radiotherapy and 5-fluorouracil [5-FU]) [100].

Heat shock protein 90 α (Hsp90α) is abundant in the cytoplasm and can form a chaperone complex with Hsp70, Hsp40, and Hop to assist in protein folding. Nonetheless, extracellular Hsp90α is reported to activate matrix metalloproteinase-2 (MMP-2) and is conducive to invasion and metastasis [101]. Gong et al. demonstrated that metformin could phosphorylate AMPKα1 and inhibit PKCγ, which suppressed the secretion of Hsp90α and invasion in the NSCLC cell line H1299 and breast cancer cell line MCF-7 but did not affect proliferation [102].

**Figure 6:**
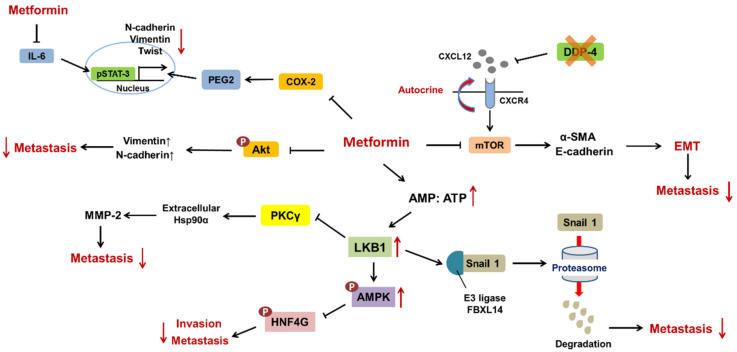
Metformin and its role in suppressing metastasis.

Another study conducted by Tong revealed that metformin could reduce CRPC metastasis through the COX-2/PEG2/STAT3 axis in the prostatic cancer cell lines PC3 and 22Rv1. Metformin blocked COX-2-mediated proinflammatory prostaglandin E2 (PGE2) generation and further suppressed the phosphorylation and nuclear translocation of p-STAT-3 but did not change the total levels of STAT3. This eventually led to decreased expression of EMT markers, including N-cadherin, vimentin and Twist, and increased levels of E-cadherin (Figure 6) [103].

The tyrosine kinase inhibitors (TKIs) erlotinib (Tarceva) and gefitinib (Iressa) are effective for patients with *EGFR*-mutant cancers, especially lung adenocarcinoma. Unfortunately, some patients develop resistance to TKIs within 10 months after administration [104,105]. Importantly, IL-6 and its downstream components, STAT3 and the proteins involved in AKT-mediated EMT, are the culprits of TKI resistance. A previous study indicated that a combination of metformin and erlotinib or gefitinib attenuated resistance in the lung adenocarcinoma gefitinib-sensitive cell line PC-9 and the gefitinib-resistant cell line PC-9GR by inhibiting the IL-6 signaling pathway. The same result could be observed in vivo in BALB/cA-nu mice inoculated with PC-9 and PC-9GR cells [106].

### 4.6. Metformin and Immunosuppression

Cancer immunosuppression has gained more attention in recent years due to remarkable breakthroughs in the development of immune checkpoint inhibitors, including anti-PD-1/PD-L1 and anti-CTLA-4 antibodies. Immunosuppression is highly correlated with cancer progression, metastasis and chemoresistance [13]. Hence, overcoming or reversing immunosuppression could promote the efficacy of cancer treatment. Metformin has been reported to play crucial roles in cancer immunity modulation. Perhaps metformin can act as a sensitizer for cancer therapies (e.g., immune checkpoint inhibitors).

#### 4.6.1. Hypoxia

**Figure 7:**
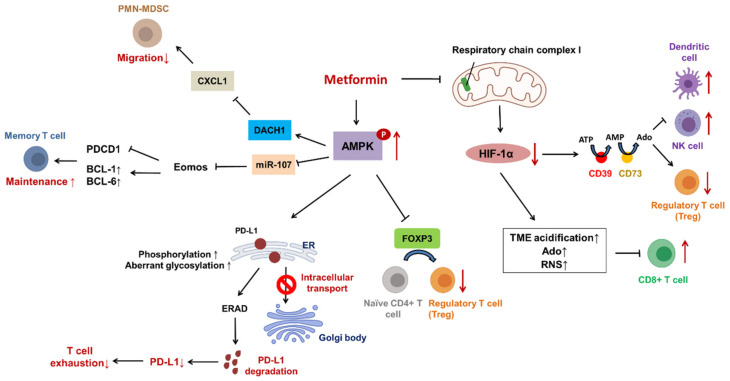
Metformin and its role in the modulation of cancer immunity.

Hypoxia serves as a suppressive factor for CD8+ T cells through several mechanisms, including tumor microenvironment (TME) acidification, extracellular accumulation of adenosine (Ado) and reactive nitrogen species (RNS). In addition, hypoxia-induced leaky blood vessels exclude CD8+ T cells from the TME. Thus, modulating hypoxic conditions can overcome resistance to immune checkpoint inhibitors (ICIs) [107,108]. Metformin was reported to impede hypoxia-mediated immunosuppression by inhibiting mitochondrial respiratory chain complex I and inducing O_2_-dependent degradation of HIF-1α in the hepatoma cell line Huh7 (Figure 7) [109]. Furthermore, Kim’s study suggested that patients with small-cell lung cancer (SCLC) had a prolonged durable response to nivolumab (FDA-approved third-line immunotherapy to SCLC) after metformin treatment to alter O_2_ consumption by nivolumab-resistant SCLC cells because the hypoxic microenvironment confers immunoresistance to cancer cells [110,111].

#### 4.6.2. PD-L1 & ICIs

Metformin is reported to exert an antitumor effect by maintaining cytotoxic T lymphocyte (CTL) functions and secreting granzyme B (Grz B) to target cells for apoptosis. Cha et al. demonstrated that metformin-induced AMPK activation leads to PD-L1 phosphorylation at S195 and then aberrant glycosylation due to mannose removal. This results in failure of intracellular transport, PD-L1 accumulation in the ER and even degradation by ER-associated protein degradation (ERAD) (Figure 7). Eventually, PD-L1 is downregulated on the surface of tumor cells, which reactivates CTLs and contributes to tumor regression in a mouse 4T1 breast tumor model [112]. Other data have shown that metformin can benefit lung cancer patients via AMPK-mediated downregulation of miR-107 and upregulation of eomesodermin (Eomes). Eomes can induce memory CD8+ T cell differentiation to central memory T (Tcm) and memory stem T (Tscm) cells by inhibiting PDCD1 expression and promoting antiapoptotic protein expression (e.g., BCL-1 and BCL-6) for the maintenance of memory T cells (Figure 7). Moreover, in vivo, metformin can increase HER-2 CAR-T efficacy in A549-bearing mice through increasing the percentages of Tcm and Tscm cells and reducing the levels of PD-1, which prevents CTL exhaustion [113]. In addition, nanotechnology has been applied in research and even clinical practice owing to some advantages, including lower toxicity to adjacent tissue and prolonged presence in the body [114,115]. MA-pepA-Ce6 nanoparticles (NPs) consist of acid-sensitive metformin-1,4-phthalaldehyde, which is cleaved under an acidic microenvironment [peptide sequences (GPLGVRGDK, pepA) and Ce6 (compound, photosensitizer)]. MMP-2 enriched in the TME can cleave pepA between glycine (G) and valine (V); subsequently, released VRGDK-Ce6 can target the integrin αvβ3 receptor and augment the efficacy of photodynamic therapy (PDT). Additionally, metformin can downregulate PD-L1 to increase sensitivity to immunotherapy. Overall, MA-pepA-Ce6 NPs can increase the effectiveness of PDT and ICIs in suppressing the growth of 4T1 mouse breast cancer cells in vitro and in vivo [116].

#### 4.6.3. Tumor-Associated Macrophages (TAMs)

TAMs are a population of macrophages located in the TME. TAMs can be classified into two subsets, including M1 and M2. In nonmalignant tumors, M1 macrophages are predominant and play a crucial role in proinflammatory and antitumor effects; conversely, M2 macrophages are alternatively activated, accumulate in malignant or advanced tumors and have anti-inflammatory, tissue repair and protumor effects [117].

Macrophage polarization refers to a process of macrophage switching between M1 and M2; therefore, it is involved in the modulation of immunity in the TME and tumor progression.

Chiang et al. suggested that metformin can induce TAM polarization to the M1 state via the indirect modulation of cytokines through the AMPK-NF-κB signaling pathway. The TNBC cell lines MDA-MB231 and MDA-MB453 were pretreated with metformin; the downregulation of M2-related cytokines, including IL-8, IL-10 and TGF-β, and upregulation of M1-related cytokines, including TNF-α and IFN-γ were observed, and these effects were accompanied by increased phosphorylation of AMPK and attenuated expression of the phosphorylated NF-κB p65 subunit. Furthermore, in vivo, after the administration of metformin, C57BL/6 mice injected with MDA-MB-231 cells showed tumor regression [118].

Another study showed that metformin treatment can switch CD68+/Arg-1+ macrophages (M2) to CD68^+^/iNOS^+^ macrophages (M1). Furthermore, a preliminary study indicated that TAMs are related to angiogenesis [119]. High-dose (300 mg/kg per day) metformin administration inhibited the expression of VEGF and FGF and impeded TAM-associated vascular architecture disorders and endothelium-generated vascular sprouting in 4T1 tumors through the modulation of macrophage polarization [120].

#### 4.6.4. Regulatory T Lymphocytes (Tregs)

CD4^+^ FOXP3^+^ Tregs, a subset of CD4+ T lymphocytes, are highly related to cancer immunosuppression [121]. Low-dose metformin cannot directly suppress cancer cell proliferation and induce apoptosis. However, low-dose but long-term (12 weeks) metformin treatment can lead to a reduction in Tregs and ESCC growth [122]. Kunisada et al. indicated that metformin can attenuate naive CD4+ T cell differentiation into Tregs via downregulation of FOXP3 in a BALB/c mouse model. FOXP3 is inhibited by metformin-mediated AMPK activation, promoting mTOR and S6 protein phosphorylation (Figure 7). Nevertheless, several studies have reported that metformin-mediated AMPK activation blocks mTOR [96,123]. Furthermore, metformin also induces metabolic reprogramming: oxidative phosphorylation switches to glycolysis with upregulation of glucose transporter (GLUT1), which suppresses the expression of IL-10 and CTLA-4 [124].

#### 4.6.5. Myeloid-Derived Suppressor Cells (MDSCs)

MDSCs are a heterogeneous population of immature immune cells responsible for T cell tolerance. They are primarily located in the blood circulation and TME and are divided into two subsets: polymorphonuclear (PMN)-MDSCs and monocytic (M)-MDSCs [125,126]. MDSCs can be recruited by chemokines and interact with corresponding receptors; these pairs include CXCL5/CXCR2 in advanced prostate cancer and CCL15/CCR1 in metastatic CRC, which exert immunosuppressive effects via several mechanisms [127,128].

MDSCs can inhibit T cell functions (e.g., secretion of IL-2) through the nitration of LCK, an important kinase for T cell activation, by RNS [129].

An experiment conducted by Qin indicated that metformin can suppress PMN-MDSC migration through AMPK-mediated upregulation of Dachshund family transcription factor 1 (DACH1) and subsequent inhibition of CXCL1. In vivo, metformin led to tumor regression owing to the decreased infiltration of PMN-MDSCs in BALB/C nude mice injected with TE7 ESCC cells (Figure 7). This seems to be a potential indicator of ESCC patient prognosis [130].

CD39 (also known as ectonucleoside triphosphate diphosphohydrolase-1, ENTPD1ase) and CD73 (also known as ecto-5′-nucleotidase, Ecto5′NTase) are ectonucleotidases expressed on the surface of tumor cells and immune cells for the metabolism of nucleotides. CD39 converts extracellular ATP into AMP; CD73 transforms extracellular AMP into Ado [131]. Extracellular Ado can inhibit the infiltration of dendritic cells (DCs) and NK cells and the production of Th1/Th2-released cytokines but enhances the proliferation of Tregs [132]. Hence, cancer cells can benefit from the accumulation of extracellular Ado in the TME, as exemplified by increased chemoresistance in NSCLC and metastasis in CRC [133,134].

Li and his colleagues revealed that the numbers of CD39+ and CD73+ MDSCs were reduced by metformin-induced downregulation of HIF-1α (Figure 7). This reversed immunosuppression in patients with ovarian cancer. Therefore, the combination of immunotherapy and metformin might extend the durable response and efficacy of ICIs [135].

## 5. Ongoing Clinical Trials of Metformin Related to Aging, DR and Cancer

In this review, the inhibitory effects of metformin on aging, DR and cancer in vitro and in vivo were comprehensively discussed above. Furthermore, we summarize in the following table the ongoing clinical trials of metformin to support this potential therapeutic strategy.


**NCT Number**

**Status**

**Phase**

**Condition/Disease**

**Description**
Anti-agingNCT02432287CompletedIVAgingEvaluate the effect of metformin on gene expression profiles in muscle and adipose tissue of older adults by RNA-SeqNCT04264897RecruitingIIIAgingAssess changes in mitochondrial function and remodeling in skeletal muscle biopsies from patients treated with or without metforminNCT01765946CompletedIVAgingEvaluate longevity-related gene expression, including SIRT1, p66Shc, p53 and mTOR, in PBMCsAnti-DRNCT02587741RecruitingIDREvaluate the efficacy of metformin on DR in comparison with Lantus and NovoMix30Anti-cancerNCT04559308RecruitingIIBreast cancer (BC)Ascertain the antitumor effect with neoadjuvant chemotherapy (e.g., paclitaxel and doxorubicin)NCT04387630RecruitingIIEarly BCEvaluate the immunostimulatory effect on preoperative chemotherapyNCT02028221Active, not recruitingIIBCInvestigate BC prevention and favorable changes in risk features, including breast density and hormone levelsNCT02488564CompletedIIOperable and locally advanced HER-2-positive BCEvaluate the antitumor effect of the combination of liposomal doxorubicin plus docetaxel, trastuzumab and metforminNCT02437656CompletedIILocally advanced rectal cancerEvaluate the antitumor effect of metformin with neoadjuvant radiochemotherapyNCT01941953CompletedIIMetastatic CRCEvaluate the efficacy of metformin plus fluorouracil in patients refractory to oxaliplatin and irinotecanNCT02614339RecruitingIIINon-DM stage II high-risk/stage III CRCDetermine the impact of additional metformin on CRC recurrenceNCT01620593CompletedIIAdvanced prostate cancerEvaluate the inhibitory effect of metformin on castration-induced tumor growth mediated by hyperinsulinemiaNCT02640534Active, not recruitingIIMetastatic castration-resistant prostate cancer (mCRPC)Determine the efficacy of metformin plus enzalutamide compared to enzalutamide alone in patients with mCRPCNCT02360618UnknownIIInvasive bladder cancerDetermine the synergistic antitumor effect of metformin and simvastatin on aggressive cancer cellsNCT02115464TerminatedIILocally advanced NSCLCEvaluate the influence of chemoradiotherapy plus metformin on progression-free survivalNCT02019979TerminatedIIStage IIIB/IV non-squamous NSCLCDetermine the effect of metformin combined with platinum-based chemotherapy in patients on a carbohydrate-restricted dietNCT01205672CompletedIEndometrial cancerEvaluate the efficacy of metformin in non-diabetic women with endometrial cancer and determine S6K expression after treatmentNCT01529593UnknownIAdvanced cancerEvaluate the efficacy of metformin plus temsirolimus (both mTOR inhibitors) on tumor regressionNCT03889795RecruitingIAdvanced pancreatic cancerEvaluate the response to and effects of C3 (simvastatin + digoxin + metformin) on disease progressionNCT02143050UnknownI/IIMetastatic melanomaAssess the safety and efficacy of metformin + dabrafenib + trametinib in patients with stage IIIC/IV melanoma

## 6. Conclusions and Perspectives

Metformin, a biguanide, has been a first-line drug for the treatment of T2DM for decades. It is reported to modulate cellular energy homeostasis by inducing the AMPK-mediated signaling pathway. Apart from its use in the treatment of diabetes, accumulating evidence has indicated the effects of metformin against aging, DR and malignancies. Aging is a biological phenomenon in all organisms and can be characterized by the accumulation of diverse deleterious changes, which brings about an increased risk of susceptibility to disease (e.g., Alzheimer’s disease, ocular diseases, immunosuppression, and cancer) and death. Metformin can modulate ROS production to extend longevity through the SIRT-3, Nrf-2/GPx7, and PRDX-2/SKN-1 signaling pathways [26,27,28]. In addition, it facilitates a prolonged life span by promoting the autophagy-mediated clearance of decaying components and suppressing mTOR-induced expression of aging-related proteins (e.g., progerin). Owing to the increased prevalence of diabetes, the incidence of DR has been rising and drawing more attention in recent years [136]. The pathogenesis and progression of DR are correlated with vascular abnormalities, retinal cell apoptosis, and senescence.

Metformin could thwart ER stress- and ROS-mediated retinal cell apoptosis via the activation of AMPK and the upregulation of MnSOD, respectively [52,56,57]. In addition, metformin circumvents NF-kB and p21 by activating SIRT-1, which results in reduced vascular permeability, angiogenesis and senescence [38,61]. Although the correlation between metformin and cancer has been established, its effects on nonapoptotic cell death, metastasis, and immunosuppression largely remain unknown. Metformin is suggested to modulate cancer progression via nonapoptotic programmed cell death mechanisms, including necroptosis, pyroptosis, and ferroptosis. Metformin is also reported to exert a negative influence on metastasis, which may occur through the inhibition of EMT-related proteins, such as α-SMA, N-cadherin, vimentin, MMP-2, and Snail. In addition, metformin is reported to restore immunosurveillance within the TME by regulating hypoxia, PD-L1 expression, TAMs, Tregs and MDSCs. This is considered a potential strategy to increase the effectiveness of immunotherapies.

Owing to the considerable amount of time and money required for novel drug approval, drug repositioning has gained more attention in recent years [70]. Hence, metformin is a potentially promising adjuvant to enhance the efficacy of first-line treatments. For instance, metformin-mediated AMPK activation contributes to PD-L1 downregulation and further T cell reactivation, indicating its potential synergistic effect with ICIs [112]. Tseng suggested that metformin reverses paclitaxel (PTX) chemoresistance in lung cancer cells via circumventing PTX-mediated MAPK-induced excision repair cross complementation 1 (ERCC1) expression [137]. In addition to its effects in combination with chemotherapy, metformin has been reported to exert antitumor effects in combination with molecular targeted therapy. Intriguingly, single-agent metformin induced proliferation via the RAS/RAF/MAPK44/42 axis in lung cancer. However, the synergistic administration of metformin and gefitinib contributed to tumor regression in mice bearing H1299 or CALU-3 GEF-R tumor xenografts through inhibition of MAPK44/42 and Akt phosphorylation [138]. Although metformin exerts antiproliferative effects by blocking mTOR, it also inhibits RAS homolog enriched in brain (Rheb), which subsequently facilitates the heterodimerization of B-RAF and C-RAF and MAPK44/42 activation. Hence, the administration of metformin combined with MEK inhibitors significantly impeded tumor progression [139]. Furthermore, Mukhopadhyay discovered reciprocal regulation between AMPK and mTOR. AMPK activation suppressed phospholipase D (PLD) activity and the levels of its product, phosphatidic acid (PA), which facilitates the formation of mTORC1 and mTORC2 complexes and further Akt activation; conversely, PLD inhibited AMPK in an mTOR-dependent manner [140]. Rapamycin is a highly specific mTOR inhibitor, but the outcomes of clinical studies have not been encouraging, primarily due to its toxicity at effective doses. In contrast, AICAR (5-aminoimidazole-4-carboxamide-1-β-4-ribofuranoside), which functions similar to metformin regarding the induction of AMPK, blocks mTOR complex 1 (mTORC1) via PLD suppression; however, this unexpectedly results in compensatory upregulation of mTORC2. Of note, AICAR was reported to enhance the efficacy of rapamycin because the AICAR-induced downregulation of PA sensitizes MDA-MB-231 cells to rapamycin by promoting apoptosis. In this respect, metformin appears to be a promising alternative that may synergize with rapamycin for cancer treatment [141].

In a phase I/II clinical study, the combination of metformin and erlotinib as second-line treatment for patients with stage IV NSCLC without EGFR mutations was evaluated, and recommended doses were identified. Most of the enrolled patients exhibited reversible GI-related adverse events, such as diarrhea and abdominal pain. Therefore, this combination regimen showed good safety in NSCLC patients; perhaps it is an applicable strategy to improve patient prognosis, but further investigation is needed [142].

The tumor suppressor effects of metformin have been well-documented [9]. Controversially, metformin may have the negative effect of accelerating cancer development. The recent in vitro and in vivo results of the study by Hampsch et al. suggested that metformin may promote the survival of dormant estrogen receptor (ER)-positive breast cancer cells by activating AMPK in a context-dependent manner [143]. Therefore, despite recent evidence supporting the anticancer and chemopreventive effects of metformin, the context-dependent effects of AMPK in cancer must be considered in the future when administering metformin as a chemoprevention or adjuvant treatment [144].

In addition, there are several urgent problems regarding the effects of metformin on cancer that require further investigation. As mentioned above, metformin induces AMPK and autophagy; nevertheless, checkpoint kinase 1 (CHK1), which is responsible for DNA repair and cell cycle arrest, is phosphorylated by AMPK signaling upon glucose deprivation, which subsequently facilitates CHK1 degradation by beta-transducin repeats-containing protein (β-TrCP) in MDA-MB-231 and H1299 cells, thereby promoting increased cell survival [145]. Deng and colleagues found that LKB1 deficiency contributes to compromised antigenic presentation in an autophagy-dependent manner. In contrast, PD-L1 mAb administration synergized with ULK1 inhibition to increase effector T cells and reduce tumor growth in an Lkb1-mutant NSCLC mouse model [146]. Furthermore, as mentioned previously, metformin induces Nrf2 and exerts antiaging and anti-DR effects. Paradoxically, metformin was reported to indirectly inhibit Nrf2 signaling. In endometrial cancer, chemoresistance to cisplatin or paclitaxel is driven by activated Nrf2 and its downstream IDH1/α-KG/TET-1/Nrf2 axis, which form a positive loop. Conversely, metformin may mitigate chemoresistance through IDH1 suppression [147]. KRAS mutations are common in PDAC patients, affecting 90%. Mukhopadhyay et al. indicated that oncogenic KRAS facilitates GEM resistance in PDAC through Nrf2 activation. Nrf2 could not only upregulate antioxidant genes but also promote glutaminolysis by increasing glutaminase 1 (GLS-1). After treatment with AI-1 to induce Nrf2 expression, the PDAC cell lines BxPC-3 and MIA PaCa-2 exhibited significant chemoresistance and poor prognosis [148].

Altogether, the accurate identification of metformin-induced signaling pathways is paramount for therapeutic applications.
